# Differential effects of thiamine and ascorbic acid in clusters of septic patients identified by latent variable analysis

**DOI:** 10.1186/s13054-024-05188-4

**Published:** 2024-11-29

**Authors:** David Legouis, Céline Monard, Aimad Ourahmoune, Sebastian Sgardello, Hervé Quintard, Gilles Criton, Frederic Sangla, Antoine Schneider

**Affiliations:** 1 Intensive Care Unit, Department of Anesthesiology, Pharmacology, Critical Care and Emergency Medicine, University Hospital of Geneva, 1205 Geneva, Switzerland; 2 Laboratory of Nephrology, Department of Physiology and Cell Metabolism, University of Geneva, 1205 Geneva, Switzerland; 3 Adult Intensive Care Unit, Centre Hospitalier Universitaire Vaudois (CHUV), Lausanne, Switzerland; 4 Department of Epidemiology and Health Systems, Quantitative Research, Center for Primary Care and Public Health (Unisanté), University of Lausanne (UNIL), Lausanne, Switzerland; 5 Medical and Quality Directorate, University Hospital and University of Geneva, 1205 Geneva, Switzerland; 6 Department of Surgery, Centre Hospitalier du Valais Romand, 1951 Sion, Switzerland; 7 Geneva School of Economics and Management, University of Geneva, 1205 Geneva, Switzerland

**Keywords:** Sepsis, Thiamine, Ascorbic acid, HAT therapy

## Abstract

**Background:**

Thiamine and ascorbic acid have been proposed to mitigate the devastating consequences of sepsis and septic shock. To date, randomized controlled trials have failed to demonstrate a benefit of these therapies and heterogeneity of treatment effect is suspected. In this study, we aimed at assessing the heterogeneity of treatment effect of thiamine (B1) and the combination of B1 plus ascorbic acid (AA + B1) in critically ill patients with sepsis.

**Methods:**

We conducted a bi-centric retrospective cohort study. All adult patients admitted to the ICU with sepsis or septic shock between January 2012 and August 2022 were included. Patient clusters were identified using latent variable analysis based on demographics and physiological variables obtained within 24 h of admission. Within each cluster and using inverse probability weighted Cox models, we compared in-hospital mortality between patients who received standard treatment (control), standard treatment plus B1 (B1 group), and standard treatment plus a combination of thiamine and ascorbic acid (AA + B1 group).

**Results:**

A total of 3465 septic patients were included, 2183, 1054 and 228 in the standard, B1 and AA + B1 groups respectively. Five clusters of patients were identified in an unsupervised manner. The “Cluster Severe” included the most severely ill patients, the “Cluster Resp” patients presented with predominantly respiratory failure, the “Cluster Old” included elderly patients with multiple comorbidities, the “Cluster Fit” patients were young, healthy with low severity indices and “Cluster Liver” included patients with predominant liver failure. B1 treatment was associated with different outcomes across the five clusters. It was associated with a lower in-hospital mortality in the “Cluster Severe” and “Cluster Resp”. On the other hand, the combination of thiamine and ascorbic acid was not associated with reduced mortality in any cluster but an increased mortality in”Cluster Old”.

**Conclusions:**

These results reinforce the lack of efficacy of the combination of AA + B1 reported in recent trials and even raise concerns about potential harm in older patients with comorbidities. On the contrary, we reported improved ICU survival associated with B1 supplementation in the most severe patients and those with predominant respiratory failure, supporting the need for further trials in this specific population.

**Supplementary Information:**

The online version contains supplementary material available at 10.1186/s13054-024-05188-4.

## Background

Sepsis is a common, life-threatening condition related to a dysregulated host response to infection leading to organ dysfunction [1–4] It is associated with an in-hospital mortality rate of 50% in patients admitted to intensive care (ICU) [1, 2, 5–8] and may be even higher in the WHO African Region [9].

To date, the management of sepsis and septic shock has relied on administration of antimicrobials, fluid resuscitation, control of the source of infection and use of vasopressors when appropriate [10–12]. Major interest in ascorbic acid (AA) and thiamine (B1) supplementation as adjunctive therapy for septic shock was triggered by the publication of a before-after observational study by Marik et al*.* that reported significant improvements in outcomes of septic patients receiving a combination of hydrocortisone, AA and B1 (HAT therapy) [13]. The authors suggest that these effects may be related to AA via several pathways. At the cellular level, AA may have antioxidant and anti-inflammatory properties. AA may also restore endothelial function and microcirculatory flow. Finally, AA is required for the synthesis of catecholamines. Thiamine was included in their protocol because high doses of ascorbic acid, combined with thiamine deficiency—common in septic ICU patients—both promote the production of oxalate, increasing the risk of tissue deposition and crystallization in the kidneys, thereby worsening renal function. However, recent randomized controlled trials (RCT) have failed to replicate these survival benefits [14–21]. A recent meta-analysis showed that the HAT therapy was only associated with a modest improvement in the duration of organ dysfunction but no survival benefit [22]. More recent studies also failed to demonstrate a mortality benefit associated with HAT therapy [23, 24].

The lack of conclusive results may be explained by heterogeneous baseline characteristics of the patients included in these trials [25]. Meaningful differences in survival may have been missed by combining patients with potential benefit, potential harm, or no effect of these treatments. More importantly, the effects of B1 or AA *plus* B1 (AA + B1) combination were never assessed separately, and unexpected interactions may counteract the combined effects, limiting the ability to assess the potential benefit of each agent separately.

In this study, we hypothesized that the addition of B1 or AA + B1 may have different effects in different clusters of septic ICU patients compared to standard treatment.

## Material and methods

### Aim of the study

The aim of this study was to compare in-hospital mortality across different clusters of septic patients based on whether they received standard treatment, standard treatment with B1 supplementation, or standard treatment with AA + B1 supplementation.

### Study design and population

In this bi-centric observational study, we considered all adult patients (≥ 18 years) admitted to the ICU of the Geneva University Hospitals (Geneva, Switzerland) and the Centre Hospitalier Universitaire Vaudois (Lausanne, Switzerland) between January 2012 and August 2022. Patients admitted with sepsis or septic shock and those with proven infection who received either mechanical ventilation or norepinephrine within 24 h of ICU admission were included. Data were extracted from electronic medical records (Metavision® (IMD Soft, Tel Aviv, Israel), Soarian® (Cerner, North Kansas City, USA), and Centricity Critical Care® (GE HealthCare, Chicago, USA).

### Treatment groups

Each patients’ cluster was divided into 3 groups according to the treatment received within the first 48 h of ICU admission. The control group included patients who received standard treatment, without B1 or AA supplementation. The B1 group included patients who received standard treatment and B1 supplementation. The AA + B1 group included patients who received standard treatment and AA + B1 supplementation.

The use of B1 and AA was not protocolized but was left to the discretion of the attending physician. Dose and duration of treatment were recorded but did not impact group assignment. They were systematically stopped at ICU discharge.

Both oral and intravenous supplementation were considered, with no minimum dose set.

### Outcomes

The primary outcome was all cause in-hospital mortality.

Ventilator-free days (VFD) at day 28 were calculated by subtracting the total days on ventilation from 28 if the patient was extubated and survived; if the patient remained on ventilation for the entire 28 days or died within the period (regardless of ventilation status), VFD was set to 0.

### Clusters of patients

Patients were clustered according to ICU admission and demographic characteristics using latent variable analysis using the Flexmix package [26]. We iteratively applied the FLXMCmvcombi model driver with 1–5 components to subgroup mixed-mode binary and Gaussian data. The optimal number of latent classes was selected to minimize the BIC criteria. The following variables were used as inputs:

- Demographic data and comorbidities (age, sex, heart failure, hypertension, chronic obstructive pulmonary disease (COPD), diabetes mellitus (DM), chronic kidney disease (CKD), chronic liver disease, body mass index (BMI)).
- Severity of illness at ICU admission (bilirubin, Aspartate Aminotransferase (AST), Alanine Aminotransferase (ALT), fibrinogen, arterial base excess, lactate, pH and serum creatinine levels, white blood cells and platelets count) and within 24 h of ICU admission (median heart rate, maximum inspired fraction of oxygen (FiO2) and minimum PaO2/FiO2 ratio).
- Need for organ support therapy within 24 h of ICU admission (cumulative dose of infused norepinephrine, renal replacement therapy (RRT) and invasive mechanical ventilation).

These variables were selected according to three criteria: (1) they should reflect patient severity, type of organ failure and comorbidities; (2) they should be recorded in both centers; (3) they should be easily identifiable by clinicians at the bedside. Composite scores were not used in order not to lose information at the level of individual variables and because these scores are rarely available to clinicians during the ICU stay, limiting their ability to recognize clusters.

A heat map was used to show the scaled means of each included variable across the identified clusters. For better visualization (e.g. increased severity associated with a higher value), excess base, pH and minimum PaO2/FiO2 ratio were shown as their opposites.

### Statistical analyses

Baseline characteristics were expressed as median and interquartile range (IQR)(25–75th percentiles) or absolute and relative (%) frequency if categorical. They were compared using a Kruskal–Wallis Rank Test or a Fisher’s Exact test depending on their class.

Rates of missing data are shown in Supplementary Fig. 1. All variables have a missing rate of less than 10%. For downstream analyses, missing data were imputed using the missForest R package [27], which uses a random forest trained on the observed values to predict the missing values. We also performed a complete cases analysis as a sensitivity analysis.

Numerical variables were centered, scaled and normalized using a Yeo-Johnson transformation, as the independent variables were on very different scales.

Within each patient subgroup, mortality was compared between treatment groups using Cox modelling. Since physicians may have treated more severe patients with the AA + B1 combination and less severe patients with standard treatment, we fitted a propensity score for receiving standard treatment, standard treatment with B1 supplementation, or standard treatment with AA + B1 supplementation. This score included the following variables.

- Hydrocortisone administration within 48 h of ICU admission.
- Demographic data and comorbidities (age, sex, BMI, Charlson comorbidities index).
- Severity of illness at ICU admission (bilirubin, arterial base excess, lactate, AST, ALT and serum creatinine levels, white blood cells count) or within the first 24 h (median heart rate, minimum PaO2/FiO2 ratio, maximum FiO2, cumulative norepinephrine dose and need for RRT).
- Year of ICU admission to account for possible changes in practice over the study period.

Within each cluster, we assessed covariate balance across the three treatment groups by calculating the absolute standardized mean difference (ASMD) both before and after propensity score weighting. An ASMD threshold of 10% after weighting was used to identify residual imbalance [28–30]. Using the propensity scores, we applied inverse probability of treatment weighting (IPTW) in a Cox proportional hazards model to estimate the treatment effect on the outcome.

For covariates where the ASMD remained above the 10% threshold after weighting, indicating persistent imbalance, we applied additional adjustments directly in the Cox model by including these covariates as independent variables. This two-step strategy, known as "double propensity score adjustment", further corrects for imbalance and refines treatment effect estimates [31, 32]. The model was then refined by retaining only the most relevant adjusting variables, selected through a backward elimination procedure.

ASMD values for each propensity score weighting scenario are presented in Supplementary Figs. 2, 4, and 5, with variables showing ASMD values exceeding 0.1 highlighted in red.

Lastly, the proportional hazards assumption was assessed for all fitted Cox models to ensure model validity [33].

All analyses were performed using R software. *P*-values were two-tailed and a value less than 0.05 was considered significant.

### Ethics

In adherence to the Swiss Federal Act on Research involving Human Beings (article 34), retrospective utilization of non-genetic health-related personal data was permitted, provided that the patient (or its legal representative) had not expressed wishes of non-participating to clinical research. This study was carried out in accordance with the principles outlined in the Declaration of Helsinki and was approved by the local ethical committee for human studies of Geneva, Switzerland (Commission Cantonal d’Ethique de la Recherche, CCER 2023-00147, “Adjunctive metabolic therapies for septic shock in ICU patients” approved November 21st 2023).

## Results

### Population

Between January 2012 and August 2022, 41,265 patients were admitted to the two participating units. Among these, 3465 patients were admitted with sepsis or septic shock and were therefore included in the study.

### Treatment groups

Among the 3465 patients included, 2183 received standard treatment (control group), 1054 received standard treatment plus B1 (B1 group), and 228 received standard treatment plus AA + B1 (AA + B1 group). Their baseline characteristics are shown in Supplementary Table 1.

Patients in the B1 and AA + B1 groups had higher disease severity compared with those in the control group. This included a higher SAPS-2 score at ICU admission, a higher cumulative 24 h norepinephrine dose, more severe lactic acidosis, and a higher proportion required mechanical ventilation or RRT within the first 24 h of ICU admission (Supplementary Table 1).

The median total cumulative received dose of B1 was 900 mg (IQR 400;2500) and 600 mg (IQR 300;1533) in the B1 and AA + B1 groups, respectively. The median total cumulative dose of AA was 1500 mg (IQR 1250;4875). The median duration of B1 treatment was 3.4 days (IQR 1;7) and 2.0 days (IQR 1;3) in the B1 and AA + B1 groups, respectively. The median duration of AA treatment was 1.2 days (IQR 1;2) (Supplementary Table 2).

### Overall treatment effect

**Fig. 1:**
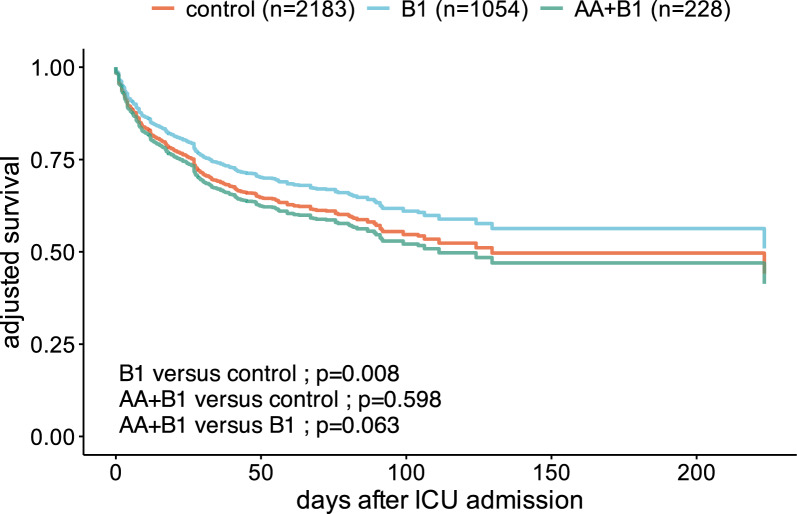
All-cause in-hospital mortality across treatment groups: Survival curves showing the cumulative hospital survival vs time across the standard treatment, B1 and AA + B1 groups, after propensity score weighting and adjustment for residual unbalanced variables

Overall in-hospital mortality was not the same across the three treatment groups, with a lower mortality observed in patients in group B1 compared to the control group (hazard ratio 0.8, 95% confidence interval [0.67;0.94], *p* = 0.008, Fig. 1 and Supplementary Fig. 2).

### Patients’ clusters

**Fig. 2:**
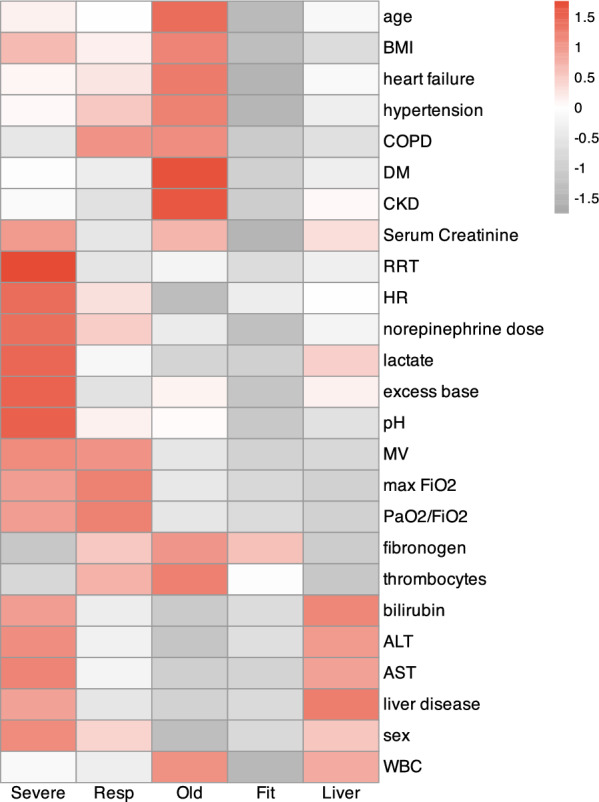
Identification of clusters of patients: Heatmap showing the scaled means of each variable used for clustering across the 5 groups identified. BMI Body Mass Index; COPD Chronic Obstructive Pulmonary Disease; DM Diabetes Mellitus; CKD Chronic Kidney Disease; RRT Renal Replacement Therapy; HR Heart Rate; MV Mechanical Ventilation; WBC White Blood Cell count, AST, Aspartate Aminotransferase; ALT Alanine Aminotransferase. For the sex variable, the highest values refer to the highest proportion of male patients. For PaO2/FiO2, base excess and pH variables, the values were inverted to improve visualization

Five clusters of patients were identified, each with specific patterns of baseline characteristics (Fig. 2). Cluster 1 included 554 patients with septic shock, multiple organ failure and high cumulative doses of norepinephrine (“Cluster Severe”). Cluster 2 included 816 patients with predominantly respiratory failure: high FiO2, low PaO2/FiO2 and limited involvement of other organs (“Cluster Resp”). Cluster 3 included 653 elderly patients with multiple comorbidities, such as CKD or COPD and a high inflammatory syndrome (“Cluster Old”). Cluster 4 included 810 patients who tended to be young, healthy and had low severity indices (“Cluster Fit”). Finally, Cluster 5 included 632 patients with predominant liver failure (“Cluster Liver”). Detailed characteristics of each subgroup are shown in Table 1.

**Table 1:** Baseline characteristics within patient clusters

	Cluster Severe (N = 554)	Cluster Resp (N = 816)	Cluster Old (N = 653)	Cluster Fit (N = 810)	Cluster Liver (N = 632)	Total (N = 3465)	*p* value
*Patients’ characteristics*							
age	68.0 (59.0, 77.0)	68.0 (58.0, 76.0)	74.0 (68.0, 81.0)	60.0 (47.0, 69.0)	66.0 (55.0, 77.0)	68.0 (57.0, 76.0)	< 0.001
Sex: Male	373 (67.3%)	534 (65.4%)	400 (61.3%)	508 (62.7%)	416 (65.8%)	2231 (64.4%)	0.157
BMI (kg/m2)	25.7 (23.0, 29.4)	25.3 (22.1, 29.1)	26.1 (23.5, 30.5)	23.9 (21.1, 26.7)	24.2 (22.3, 27.2)	24.8 (22.3, 28.4)	< 0.001
saps2	73.0 (63.0, 86.0)	55.5 (45.0, 68.0)	51.0 (42.0, 61.0)	38.0 (29.0, 48.0)	49.0 (40.0, 61.0)	52.0 (40.0, 66.0)	< 0.001
Charlson score	4.0 (2.0, 6.0)	3.0 (2.0, 6.0)	4.0 (2.0, 6.0)	2.0 (0.0, 5.0)	4.0 (2.0, 6.0)	3.0 (2.0, 6.0)	< 0.001
Year of ICU admission	2016.(2013, 2018)	2015 (2013, 2018)	2015 (2013,2018)	2016 (2013,2018)	2015 (2013,2018)	2015 (2013,2018)	0.089
*Underlying diseases*							
CKD	130 (23.5%)	127 (15.6%)	338 (51.8%)	74 (9.1%)	162 (25.6%)	831 (24.0%)	< 0.001
diabetes	106 (19.1%)	140 (17.2%)	198 (30.3%)	109 (13.5%)	108 (17.1%)	661 (19.1%)	< 0.001
hypertension	216 (39.0%)	366 (44.9%)	354 (54.2%)	162 (20.0%)	213 (33.7%)	1311 (37.8%)	< 0.001
heart_failure	111 (20.0%)	172 (21.1%)	186 (28.5%)	77 (9.5%)	118 (18.7%)	664 (19.2%)	< 0.001
chronic liver disease	93 (16.8%)	56 (6.9%)	26 (4.0%)	43 (5.3%)	121 (19.1%)	339 (9.8%)	< 0.001
copd	46 (8.3%)	127 (15.6%)	105 (16.1%)	48 (5.9%)	49 (7.8%)	375 (10.8%)	< 0.001
*At ICU admission*							
pH	7.2 (7.1, 7.3)	7.3 (7.3, 7.4)	7.3 (7.3, 7.4)	7.4 (7.4, 7.5)	7.4 (7.3, 7.4)	7.4 (7.3, 7.4)	< 0.001
Base excess (mmol/L)	− 11.8 (− 14.8, − 9.0)	− 4.3 (− 7.0, − 1.4)	− 6.5 (− 9.8, − 3.6)	− 2.3 (− 4.7, 0.8)	− 6.7 (− 9.1, − 4.1)	− 5.6 (− 9.2, − 2.1)	< 0.001
HCO3 (mmol/L)	15.1 (12.4, 17.9)	20.9 (18.4, 23.9)	18.5 (15.9, 21.7)	21.8 (19.3, 24.8)	17.8 (15.5, 20.4)	19.2 (16.3, 22.5)	< 0.001
Lactate (mmol/L)	5.4 (3.4, 8.0)	1.8 (1.2, 2.8)	1.2 (0.8, 2.0)	1.2 (0.9, 1.8)	2.6 (1.7, 4.0)	1.8 (1.1, 3.3)	< 0.001
Bilirubine (mol/L)	25.0 (13.0, 55.0)	13.0 (10.0, 20.0)	10.0 (6.0, 16.0)	12.0 (8.0, 18.0)	27.0 (14.0, 58.0)	14.0 (9.0, 26.9)	< 0.001
AST (UI/L)	155 (68, 377)	45 (29, 73)	27 (20, 42)	33 (20, 51)	106 (59, 193)	48 (27, 101)	< 0.001
ALT (UI/L)	70.5 (35.0, 204.2)	29.0 (18.0, 51.2)	18.0 (13.0, 27.0)	25.0 (15.0, 43.0)	61.0 (36.5, 125.2)	32.0 (18.0, 62.7)	< 0.001
Fibrinogen (g/L)	3.7 (2.4, 4.8)	4.9 (3.8, 6.3)	5.3 (4.4, 6.4)	4.9 (4.1, 6.0)	3.8 (2.4, 5.0)	4.7 (3.4, 5.9)	< 0.001
Thrombocytes (G/L)	126 (64, 201)	198 (127, 272)	214 (161, 299)	173 (87, 259)	116 (69, 171)	169 (99, 250)	< 0.001
Serum creatinine (µmol/L)	205 (147, 284)	101 (73; 144)	169 (114, 281)	70 (54, 89)	148 (104, 206)	121 (78, 196)	< 0.001
WBC counts (G/L)	12.7 (5.2, 20.1)	11.9 (6.3, 17.8)	14.1 (9.8, 19.4)	10.5 (5.0, 15.5)	13.9 (7.8, 23.5)	12.4 (6.9, 18.7)	< 0.001
*Within 24 h following ICU admission*							
Maximal FiO2 (%)	99.9 (68.9, 100.0)	99.9 (75.0, 100.0)	41.4 (31.0, 59.5)	37.2 (28.0, 50.0)	35.0 (28.0, 45.0)	50.6 (35.0, 93.3)	< 0.001
Minimal PaO2/FiO2 ratio	118 (80; 182)	116 (80, 161)	219 (160, 279)	231 (172, 288)	244 (194, 301)	187.0 (122, 260)	< 0.001
Maximal PEEP (cmH20)	8.0 (6.4, 10.0)	8.0 (6.6, 10.0)	6.7 (5.0, 8.0)	6.0 (5.0, 7.8)	6.1 (5.0, 7.1)	7.0 (5.1, 9.0)	< 0.001
Cum norepinephrine (mg)	34.0 (17.8, 59.2)	12.3 (4.3, 24.0)	4.0 (0.5, 10.0)	0.3 (0.0, 2.9)	5.2 (0.7, 12.3)	5.8 (0.7, 18.1)	< 0.001
RRT	238 (43.0%)	23 (2.8%)	50 (7.7%)	0 (0.0%)	38 (6.0%)	349 (10.1%)	< 0.001
MV	447 (80.7%)	616 (75.5%)	192 (29.4%)	152 (18.8%)	130 (20.6%)	1537 (44.4%)	< 0.001
Hydrocortisone	441 (79.6%)	410 (50.2%)	250 (38.3%)	160 (19.8%)	275 (43.5%)	1536 (44.3%)	< 0.001
*Treatments group*							
control	242 (43.7%)	437 (53.6%)	491 (75.2%)	619 (76.4%)	394 (62.3%)	2183 (63.0%)	< 0.001
B1	245 (44.2%)	312 (38.2%)	127 (19.4%)	167 (20.6%)	203 (32.1%)	1054 (30.4%)	
AA + B1	67 (12.1%)	67 (8.2%)	35 (5.4%)	24 (3.0%)	35 (5.5%)	228 (6.6%)	
*Outcomes*							
ICU LOS (days)	5.5 (1.5, 12.6)	6.5 (3.2, 11.8)	2.8 (1.6, 4.9)	2.1 (1.1, 3.9)	2.7 (1.6, 5.1)	3.3 (1.6, 7.5)	< 0.001
Hospital LOS (days)	10.0 (2.0, 30.6)	21.9 (10.0, 36.9)	18.0 (10.0, 33.1)	17.0 (9.0, 32.2)	15.0 (6.6, 28.6)	17.0 (7.7, 33.0)	< 0.001
							< 0.001
No AKI	212 (38.3%)	330 (40.4%)	418 (64.0%)	569 (70.2%)	362 (57.3%)	1891 (54.6%)	
KDIGO1	25 (4.5%)	94 (11.5%)	59 (9.0%)	97 (12.0%)	74 (11.7%)	349 (10.1%)	
KDIGO2	54 (9.7%)	219 (26.8%)	96 (14.7%)	111 (13.7%)	100 (15.8%)	580 (16.7%)	
KDIGO 3	263 (47.5%)	173 (21.2%)	80 (12.3%)	33 (4.1%)	96 (15.2%)	645 (18.6%)	
VFD at day28	0.0 (0.0, 18.3)	20.1 (0.0, 25.0)	25.2 (12.5, 27.1)	25.8 (19.5, 27.3)	23.3 (0.0, 26.8)	20.8 (0.0, 26.0)	< 0.001
ICU mortality (%)	285 (51.4%)	133 (16.3%)	37 (5.7%)	47 (5.8%)	86 (13.6%)	588 (17.0%)	< 0.001
Hospital mortality (%)	316 (57.0%) 5 missing	192 (23.5%) 25 missing	99 (15.2%) 39 missing	107 (13.2%) 63 missing	145 (22.9%) 49 missing	859 (24.8%) 181 missing	< 0.001
28 days mortality	309 (56.3%)	167 (21.1%)	91 (14.8%)	93 (12.4%)	137 (23.5%)	797 (24.3%)	< 0.001

*BMI* Body mass index, *SAPSII* Simplified acute physiological score, *MV* Mechanical ventilation, *WBC* White blood cells, *RRT* Renal replacement therapy, *ICU LOS* Intensive care unit length of stay, *AKI* Acute kidney injury, *AST* Aspartate aminotransferase, *ALT* Alanine aminotransferase, *VFD* Ventilator free days, *CKD* Chronic kidney disease

Data were shown as number (%) or median (IQR)

All-cause in-hospital mortality was different across the clusters and ranged from 13% in «Cluster Fit» to 57% in “Cluster Severe” (Table 1).

### Treatment regimen across clusters

The cumulative dose of thiamine was not evenly distributed across clusters and treatment groups. Specifically, patients in group B1 and “Cluster Severe”, as well as patients from “Cluster Resp” also in group B1, received significantly more thiamine than others (1200 (IQR 500;3000) and 800 mg (IQR 300;1800), respectively, *p* < 0.001, Supplementary Fig. 3a and Supplementary Table 2 and 3).

For ascorbic acid, there was no significant difference in the cumulative dose received between clusters Supplementary Fig. 3b and Supplementary Table 2 and 3).

### Treatment effect across clusters

**Fig. 3:**
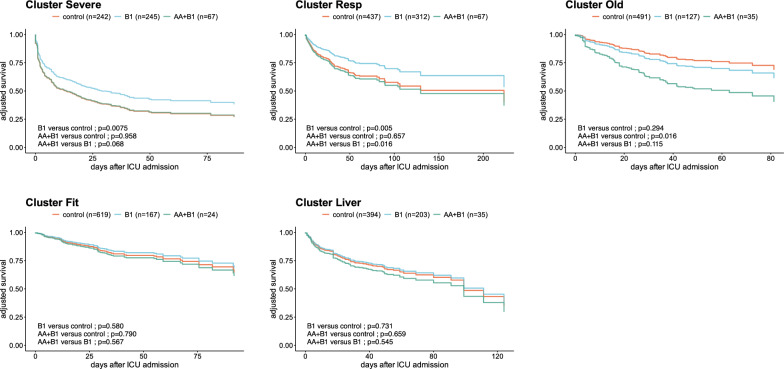
All-cause in-hospital mortality across clusters of patients: Survival curves showing the cumulative hospital survival vs time across the standard treatment, B1 and AA + B1 groups for each cluster of patients, after propensity score weighting and adjustment for residual unbalanced variables

In the “Cluster Severe” and “Cluster Resp”, B1 supplementation was associated with improved survival (HR for mortality 0.71 [0.56;0.91], *p* = 0.008 and 0.60 [0.42;0.86], *p* = 0.005, respectively), while AA + B1 supplementation was not. In “Cluster Old”, AA + B1 treatment was associated with harm (HR for mortality 2.77 [1.21,6.36], *p* = 0.016). Finally, in “Cluster Fit” and “Cluster Liver”, there was no difference between treatment groups (Fig. 3, Supplementary Fig. 4).

### Sensitivity analyses

First, we assessed whether our results might have changed if we had considered ICU mortality instead of in-hospital mortality. We therefore used the same strategy as before, fitting a propensity score to perform weighted Cox modelling, with further adjustment for variables that were still imbalanced. Regarding ICU hospital mortality, B1 supplementation was associated with improved survival in "Cluster Severe" and "Cluster Resp" (HR for ICU mortality 0.7 [0.5;0.9], *p* = 0.011 and 0.5 [0.3;0.8], *p* = 0.002, respectively), whereas AA + B1 supplementation was not. In the “Cluster Old”, AA + B1 treatment was associated with harm (HR for mortality 2.8 [1.7.5], *p* = 0.048). Finally, there was no statistically significant difference between treatment groups in "Cluster Fit" and "Cluster Liver".

Second, we examined whether a center effect might have added bias to the results. We therefore included center as an interaction term with the treatment group in the Cox model. Within the 5 clusters identified, no interaction had a *p*-value below 0.05, making the existence of a center effect unlikely.

Finally, we reassessed all-cause in-hospital mortality in the three treatment groups from the dataset excluding patients with missing values. We found similar results, with a trend towards to lower mortality observed in patients in group B1 compared with the other two groups (hazard ratio 0.83, 95% confidence interval [0.69;1.01], *p* = 0.061, Supplementary Fig. 5).

## Discussion

In this study, we performed unsupervised clustering and identified five clusters of septic patients corresponding to meaningful clinical situations. We then assessed the association between standard treatment, B1 or AA + B1 treatment and in-hospital mortality in each of these clusters. We found that the B1 treatment was associated with improved survival in patients with a high level of severity and those with severe respiratory failure (“Cluster Severe” and “Cluster Resp”) but not in the other clusters. In contrast, AA + B1 treatment was not associated with a survival benefit in any cluster and was actually seen to be harmful in older patients with comorbidities (“Cluster Old”).

While recent RCTs have focused on B1 in combination with AA [14–21], little is known on the effects of B1 alone on outcomes in septic patients. Small trials have suggested a potential benefit. Recently in a small RCT, Nandhini et al., found a reduction in ICU mortality in the group treated with B1 alone (2 mg/kg/8 h) compared to the groups treated with AA alone (50 mg/kg/6 h) or placebo (respectively 28%, 48% and 60%) [34]. A study conducted by Donnino et al., enrolling 88 septic shock patients, compared intravenous infusion of B1 (200 mg/12 h) to a placebo. In the predefined subgroup of patients with B1 deficiency, mortality was reduced in the intervention group (13 versus 46%, *p* = 0.047 for survival analyses) [35]. Finally, a greater reduction in the vasopressor dependency index in patients treated with B1 was observed in a similar study conducted by Petsakul et al., in 50 ICU patients with septic shock [36]. From a biological point of view, there is a rationale for B1 supplementation in septic shock. B1 acts as a cofactor for the pyruvate dehydrogenase (PDH) complex and alpha-ketoglutarate dehydrogenase. Both enzymes are involved in the tricarboxylic acid cycle, making thiamine necessary for mitochondrial function and ATP production [37]. Our group has also reported its role in renal gluconeogenesis, a process whose decline is associated with mortality in critically ill patients [38]. In addition, low B1 serum levels are observed in 20–70% of ICU patients [35] and are associated with mortality [39]. In a meta-analysis of RCTs assessing the effect of B1 versus monotherapy in patients with septic shock, we found a trend towards a reduction in mortality, although the difference was not statistically significant [40].

Interestingly, the two clusters in which B1 was associated with improved survival were also the clusters in which patients received the highest dose of thiamine, similar to the studies discussed above [34–36]. However, a possible threshold effect has never evaluated.

Surprisingly, we found that AA + B1 supplementation was consistently associated with similar or, in the “Cluster Old”, worse outcomes than control or B1 groups. This is unexpected because one of the cornerstones of the HAT therapy has been the presumed positive synergistic effect between its components [13] although this has never been proven. These results may raise concerns about the potential toxicity of ascorbic acid, which may have counteracted the beneficial effect of thiamine supplementation. This is consistent with the recent RCTs evaluating the effect of the combination of hydrocortisone, ascorbic acid and thiamine, all of which failed to show a benefit [14–21]. In addition, the increased mortality observed in the AA group from "Cluster Old" may also reveal a specific population in which ascorbic acid would be particularly harmful. This hypothesis may be supported by a large and recent multicenter study. Indeed, in a pre-specified subgroup analysis of the LOVIT trial, an 872-patient RCT comparing AA with placebo in septic shock, AA was associated with increased death or persistent organ dysfunction in older patients, women and those with lower severity indices [41]. This population appears to be largely similar to our “Cluster Old”, where AA + B1 was associated with higher mortality. Similarly, a large RCT of hospitalized patients with COVID-19, including patients with similar rates of comorbidities than our”Cluster Old”, demonstrated high posterior probabilities (> 90% for organ support–free days and > 75% for hospital survival) that AA had a negative impact on both outcomes in both critically and non-critically ill patients [42].

Our study has several strengths. We used unsupervised clustering to identify patient clusters. Unsupervised clustering is often challenged by the clinical relevance of the identified clusters and their relevance at the bedside. In our study, latent variable analyses identified clusters of patients with easily identifiable clinical contexts. These five clusters, corresponding to five different clinical situations, are well recognized in the ICU setting and should be easily transferable to other datasets. In addition, variables used as input were available within the first 24 h of ICU admission, allowing very early recognition of the patient’s phenotype. Furthermore, these analyses provided new insight into the role of B1 and AA as adjunctive therapies for sepsis. Finally, we used propensity-score weighting and additional adjustment for variables with residual imbalance in our Cox model to reduce the imbalance in confounders.

Our study has several limitations. Firstly, there are several potential ways to classify patients using unsupervised learning [43]. Even in the context of LCA, classification relies on the selection of class-defining variables, the number of classes and the model parameters. Although we selected variables a priori and with clinical emphasis, classifying patients using a different combination of clinical data, biomarkers, and other types of data may have triggered different classifications. However, the identified clusters appear to be associated with relevant clinical situations. Secondly, this is a retrospective study, and not all laboratory measurements were available for all patients, and some were imputed but sensitivity analyses conducted after exclusion of patients with missing values are in line with our main results. Thirdly, in the absence of a standardized dose, our results may be biased by the different dose of B1 across subgroups. Fourthly, the proportion of patients in the treatment groups was not homogeneous across clusters, with some groups having limited sample size. This may have reduced the statistical power of the Cox model, limiting the ability to identify a significant association. Fifthly, our cohort did not include patients supplemented with ascorbic acid alone. This group could have provided additional information on possible antagonism between ascorbic acid and thiamine supplementation or independent ascorbic acid toxicity in this population. Sixthly, data on treatment limitations were not collected in this cohort, and we cannot distinguish mortality due to treatment discontinuation from other causes of mortality. Finally, the most severe patients were more likely to receive AA + B1 treatment. Although we applied inverse probability of treatment weighting in Cox modelling and additional adjustments for residual imbalances, we cannot exclude the possibility of residual unmeasured confounders between groups. However, the B1 group also included patients with greater severity than the control group, yet we observed an improvement in survival within this group.

This study is hypothesis-generating by nature. Therefore, the validity of our results would ultimately need to be assessed in a prospective setting.

## Conclusion

We found that B1 administration within 48 h of ICU admission was associated with different outcomes across different clusters of septic patients with some (respiratory failure and those with the highest severity) showing a potential benefit. On the other hand, the combination of AA and B1, was not associated with benefit in any of the subgroups and was even associated with increased mortality in older patients with comorbidities. These results support the need for further large clinical trials to assess the effect of B1 supplementation in especially in septic patients with severe disease or with predominantly pulmonary involvement.

## Supplementary Information

Additional file1 (PDF 357 kb)

Additional file2 (DOCX 25 kb)

## Data Availability

The datasets used and/or analyzed during the current study are available from the corresponding author on reasonable request.

## References

[1] Singer M, Deutschman CS, Seymour CW, et al. The third international consensus definitions for sepsis and septic shock (Sepsis-3). JAMA. 2016;315(8):801. 26903338 10.1001/jama.2016.0287 PMC4968574

[2] Vincent J-L, Sakr Y, Sprung CL, et al. Sepsis in European intensive care units: results of the SOAP study. Crit Care Med. 2006;34(2):344–53. 16424713 10.1097/01.ccm.0000194725.48928.3a

[3] Xie J, Wang H, Kang Y, et al. The epidemiology of sepsis in chinese ICUs: a national cross-sectional survey. Crit Care Med. 2020;48(3):e209–18. 31804299 10.1097/CCM.0000000000004155

[4] Vincent J-L, Marshall JC, Namendys-Silva SA, et al. Assessment of the worldwide burden of critical illness: the intensive care over nations (ICON) audit. Lancet Respir Med. 2014;2(5):380–6. 24740011 10.1016/S2213-2600(14)70061-X

[5] Sakr Y, Jaschinski U, Wittebole X, et al. Sepsis in intensive care unit patients: worldwide data from the intensive care over nations audit. Open Forum Infect Dis. 2018;5(12):ofy313. 30555852 10.1093/ofid/ofy313 PMC6289022

[6] Fleischmann C, Scherag A, Adhikari NKJ, et al. Assessment of global incidence and mortality of hospital-treated sepsis. Current estimates and limitations. Am J Respir Crit Care Med. 2015;193(3):259–72. 10.1164/rccm.201504-0781OC 26414292

[7] Kaukonen K-M, Bailey M, Pilcher D, Cooper DJ, Bellomo R. Systemic inflammatory response syndrome criteria in defining severe sepsis. N Engl J Med. 2015;372(17):150319144911000. 10.1056/NEJMoa1415236 25776936

[8] Dombrovskiy VY, Martin AA, Sunderram J, Paz HL. Rapid increase in hospitalization and mortality rates for severe sepsis in the United States: a trend analysis from 1993 to 2003. Crit Care Med. 2007;35(5):1244–50. 17414736 10.1097/01.CCM.0000261890.41311.E9

[9] Fleischmann-Struzek C, Mellhammar L, Rose N, et al. Incidence and mortality of hospital- and ICU-treated sepsis: results from an updated and expanded systematic review and meta-analysis. Intensive Care Med. 2020;46(8):1552–62. 32572531 10.1007/s00134-020-06151-x PMC7381468

[10] Howell MD, Davis AM. Management of sepsis and septic shock. JAMA. 2017;317(8):847. 28114603 10.1001/jama.2017.0131

[11] Yealy DM, Mohr NM, Shapiro NI, Venkatesh A, Jones AE, Self WH. Early care of adults with suspected sepsis in the emergency department and out-of-hospital environment: a consensus-based task force report. Ann Emerg Med. 2021;78(1):1–19. 33840511 10.1016/j.annemergmed.2021.02.006

[12] Evans L, Rhodes A, Alhazzani W, et al. Surviving sepsis campaign: international guidelines for management of sepsis and septic shock 2021. Intensive Care Med. 2021;47(11):1181–247. 34599691 10.1007/s00134-021-06506-y PMC8486643

[13] Marik PE, Khangoora V, Rivera R, Hooper MH, Catravas J. Hydrocortisone, vitamin C, and thiamine for the treatment of severe sepsis and septic shock: a retrospective before-after study. Chest. 2017;151(6):1229–38. 27940189 10.1016/j.chest.2016.11.036

[14] Fujii T, Luethi N, Young PJ, et al. Effect of vitamin C, hydrocortisone, and thiamine vs hydrocortisone alone on time alive and free of vasopressor support among patients with septic shock: the VITAMINS randomized clinical trial. JAMA. 2020;323(5):423–31. 31950979 10.1001/jama.2019.22176 PMC7029761

[15] Iglesias J, Vassallo AV, Patel VV, Sullivan JB, Cavanaugh J, Elbaga Y. Outcomes of metabolic resuscitation using ascorbic acid, thiamine, and glucocorticoids in the early treatment of sepsis: the ORANGES trial. Chest. 2020;158(1):164–73. 32194058 10.1016/j.chest.2020.02.049

[16] Mohamed ZU, Prasannan P, Moni M, et al. Vitamin c therapy for routine care in septic shock (ViCTOR) trial: effect of intravenous vitamin C, thiamine, and hydrocortisone administration on inpatient mortality among patients with septic shock. Indian J Crit Care Med. 2020;24(8):653–61. 33024370 10.5005/jp-journals-10071-23517 PMC7519616

[17] Moskowitz A, Huang DT, Hou PC, et al. Effect of ascorbic acid, corticosteroids, and thiamine on organ injury in septic shock: the ACTS randomized clinical trial. JAMA. 2020;324(7):642–50. 32809003 10.1001/jama.2020.11946 PMC7435341

[18] Reddy PR, Samavedam S, Aluru N, Yelle S, Rajyalakshmi B. Metabolic resuscitation using hydrocortisone, ascorbic acid, and thiamine: do individual components influence reversal of shock independently? Indian J Crit Care Med. 2020;24(8):649–52. 33024369 10.5005/jp-journals-10071-23515 PMC7519600

[19] Wani SJ, Mufti SA, Jan RA, et al. Combination of vitamin C, thiamine and hydrocortisone added to standard treatment in the management of sepsis: results from an open label randomised controlled clinical trial and a review of the literature. Infect Dis (Lond). 2020;52(4):271–8. 31990246 10.1080/23744235.2020.1718200

[20] Chang P, Liao Y, Guan J, et al. Combined treatment with hydrocortisone, vitamin C, and thiamine for sepsis and septic shock: a randomized controlled trial. Chest. 2020;158(1):174–82. 32243943 10.1016/j.chest.2020.02.065

[21] Sevransky JE, Rothman RE, Hager DN, et al. Effect of vitamin C, thiamine, and hydrocortisone on ventilator- and vasopressor-free days in patients with sepsis: the VICTAS randomized clinical trial. JAMA. 2021;325(8):742. 33620405 10.1001/jama.2020.24505 PMC7903252

[22] Assouline B, Faivre A, Verissimo T, et al. Thiamine, ascorbic acid, and hydrocortisone as a metabolic resuscitation cocktail in sepsis: a meta-analysis of randomized controlled trials with trial sequential analysis. Crit Care Med. 2021;49(12):2112–20. 34582409 10.1097/CCM.0000000000005262

[23] Lyu Q-Q, Zheng R-Q, Chen Q-H, Yu J-Q, Shao J, Gu X-H. Early administration of hydrocortisone, vitamin C, and thiamine in adult patients with septic shock: a randomized controlled clinical trial. Crit Care. 2022;26(1):295. 36171582 10.1186/s13054-022-04175-x PMC9520942

[24] Hussein AA, Sabry NA, Abdalla MS, Farid SF. A prospective, randomised clinical study comparing triple therapy regimen to hydrocortisone monotherapy in reducing mortality in septic shock patients. Int J Clin Pract. 2021;75(9):e14376. 34003568 10.1111/ijcp.14376

[25] Iwashyna TJ, Burke JF, Sussman JB, Prescott HC, Hayward RA, Angus DC. Implications of heterogeneity of treatment effect for reporting and analysis of randomized trials in critical care. Am J Respir Crit Care Med. 2015;192(9):1045–51. 26177009 10.1164/rccm.201411-2125CP PMC4642199

[26] Leisch F. FlexMix: A General Framework for Finite Mixture Models and Latent Class Regression in R. J Stat Soft, 2004, 11(8). Available from: http://www.jstatsoft.org/v11/i08/

[27] Stekhoven DJ, Bühlmann P. MissForest–non-parametric missing value imputation for mixed-type data. Bioinformatics. 2012;28(1):112–8. 22039212 10.1093/bioinformatics/btr597

[28] Austin PC. Balance diagnostics for comparing the distribution of baseline covariates between treatment groups in propensity-score matched samples. Stat Med. 2009;28(25):3083–107. 19757444 10.1002/sim.3697 PMC3472075

[29] Stuart EA, Lee BK, Leacy FP. Prognostic score–based balance measures for propensity score methods in comparative effectiveness research. J Clinic Epidemiol. 2013;66(80):S84. 10.1016/j.jclinepi.2013.01.013 PMC3713509 23849158

[30] Zhang Z, Kim HJ, Lonjon G, Zhu Y. Group written on behalf of AB-DCTC. Balance diagnostics after propensity score matching. Annal Translat Med. 2019;7(1):16. 10.21037/atm.2018.12.10 PMC6351359 30788363

[31] Austin PC. Double propensity-score adjustment: a solution to design bias or bias due to incomplete matching. Stat Methods Med Res. 2014;26(1):201–22. 10.1177/0962280214543508 PMC5302082 25038071

[32] Nguyen T-L, Collins GS, Spence J, et al. Double-adjustment in propensity score matching analysis: choosing a threshold for considering residual imbalance. BMC Med Res Methodol. 2017;17(1):78. 28454568 10.1186/s12874-017-0338-0 PMC5408373

[33] Grambsch PM, Therneau TM. Proportional hazards tests and diagnostics based on weighted residuals. Biometrika. 1994;81(3):515–26.

[34] Nandhini N, Malviya D, Parashar S, Pandey C, Nath SS, Tripathi M. Comparison of the effects of vitamin C and thiamine on refractory hypotension in patients with sepsis: a randomized controlled trial. Int J Crit Illn Inj Sci. 2022;12(3):138–45. 36506923 10.4103/ijciis.ijciis_107_21 PMC9728072

[35] Donnino MW, Andersen LW, Chase M, et al. Randomized, double-blind, placebo-controlled trial of thiamine as a metabolic resuscitator in septic shock: a pilot study. Crit Care Med. 2016;44(2):360–7. 26771781 10.1097/CCM.0000000000001572 PMC4754670

[36] Petsakul S, Morakul S, Tangsujaritvijit V, Kunawut P, Singhatas P, Sanguanwit P. Effects of thiamine on vasopressor requirements in patients with septic shock: a prospective randomized controlled trial. BMC Anesthesiol. 2020;20(1):280. 33167911 10.1186/s12871-020-01195-4 PMC7650202

[37] Depeint F, Bruce WR, Shangari N, Mehta R, O’Brien PJ. Mitochondrial function and toxicity: role of B vitamins on the one-carbon transfer pathways. Chem Biol Interact. 2006;163(1–2):113–32. 16814759 10.1016/j.cbi.2006.05.010

[38] Legouis D, Ricksten S-E, Faivre A, et al. Altered proximal tubular cell glucose metabolism during acute kidney injury is associated with mortality. Nat Metab. 2020;2(8):732–43. 32694833 10.1038/s42255-020-0238-1

[39] Cruickshank AM, Telfer AB, Shenkin A. Thiamine deficiency in the critically ill. Intensive Care Med. 1988;14(4):384–7. 3136196 10.1007/BF00262893

[40] Sangla F, Verissimo T, Faivre A, et al. Thiamine as a metabolic resuscitator in septic shock: a meta-analysis of randomized controlled trials with trial sequential analysis. Front Med (Lausanne). 2023;10:1223862. 37780556 10.3389/fmed.2023.1223862 PMC10533915

[41] Lamontagne F, Masse M-H, Menard J, et al. Intravenous vitamin C in adults with sepsis in the intensive care unit. N Engl J Med. 2022;386(25):2387–98. 35704292 10.1056/NEJMoa2200644

[42] LOVIT-COVID Investigators, on behalf of the Canadian critical care trials group, and the REMAP-CAP investigators, Adhikari NKJ, Hashmi M, et al. intravenous vitamin C for patients hospitalized with COVID-19: two harmonized randomized clinical trials. *JAMA* 2023; 330(18):1745–1759 10.1001/jama.2023.21407 PMC10600726 37877585

[43] Castela Forte J, Perner A, van der Horst ICC. The use of clustering algorithms in critical care research to unravel patient heterogeneity. Intensive Care Med. 2019;45(7):1025–8. 31062051 10.1007/s00134-019-05631-z

