# Supplementary material for: Differential effects of thiamine and ascorbic acid in clusters of septic patients identified by latent variable analysis
Source: Crit Care. 2024 Nov 29;28:396. doi: 10.1186/s13054-024-05188-4 (PMC11606082; doi:10.1186/s13054-024-05188-4)

Supplementary Figure 1

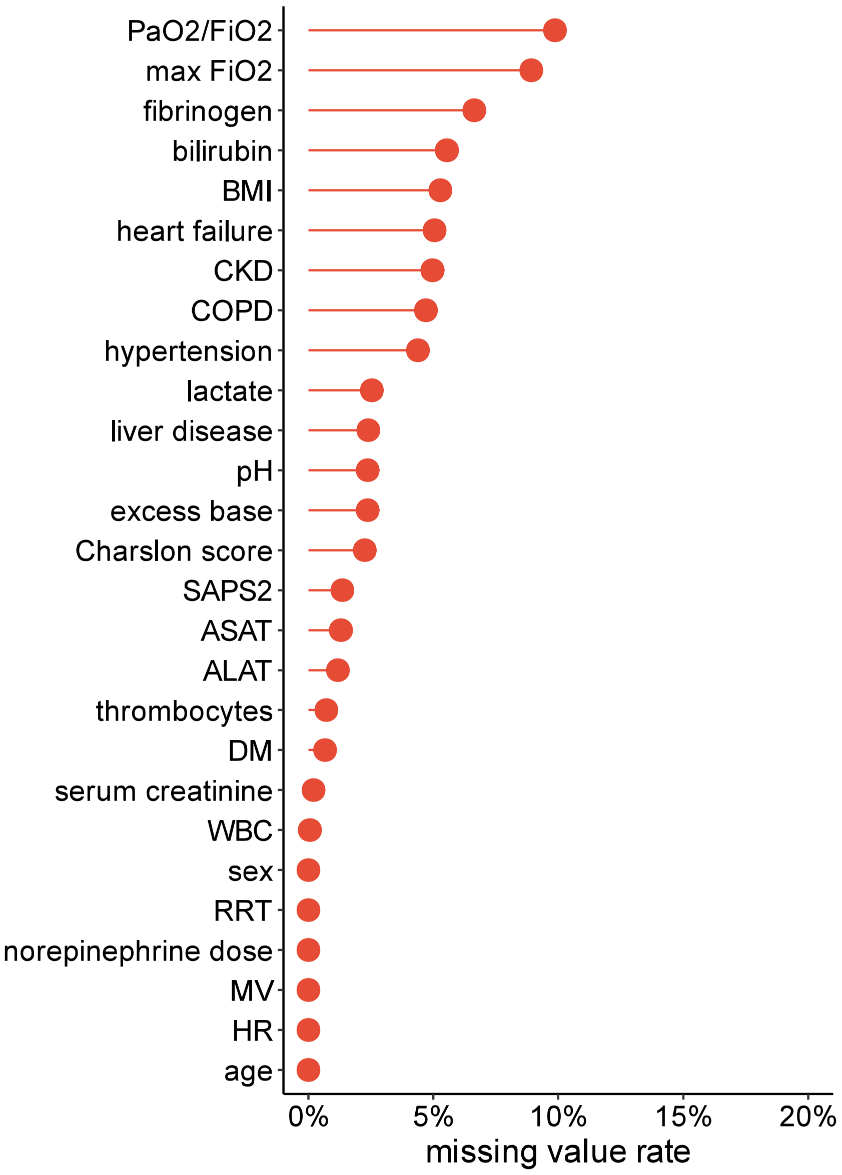

Supplementary Figure 2

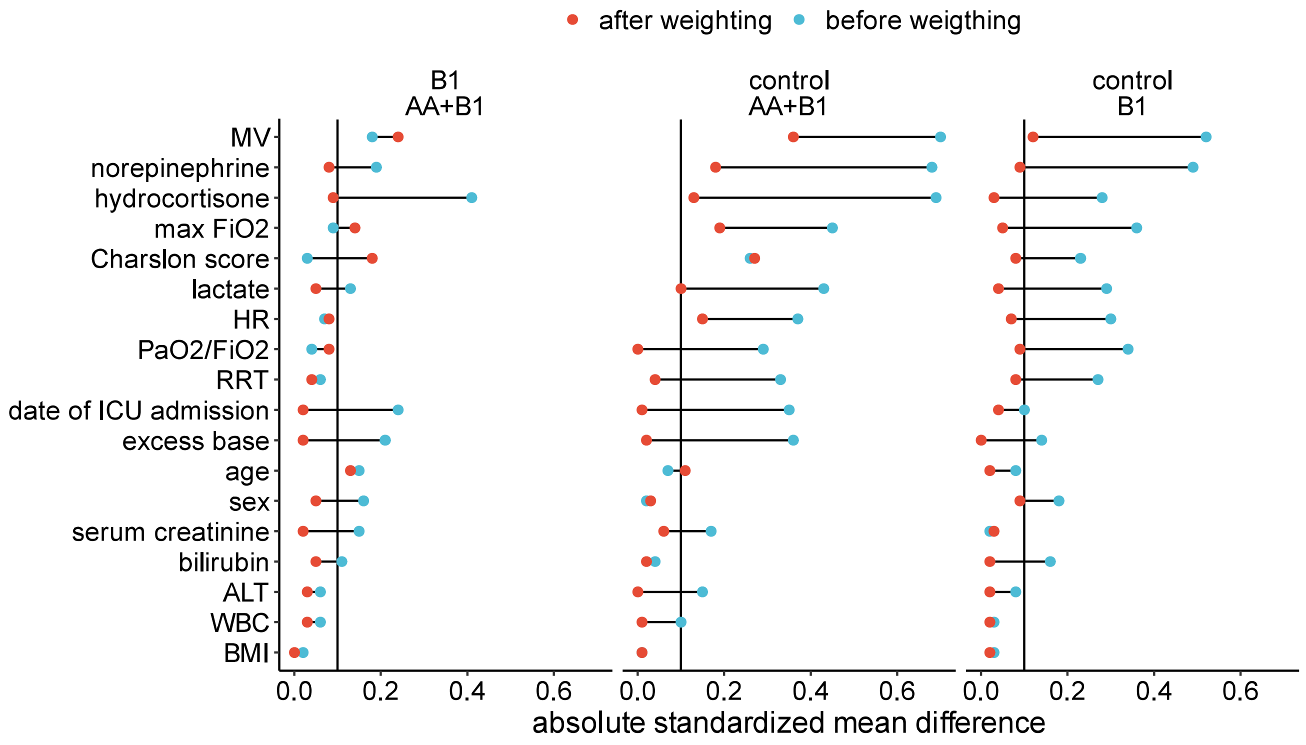

Supplementary Figure 3

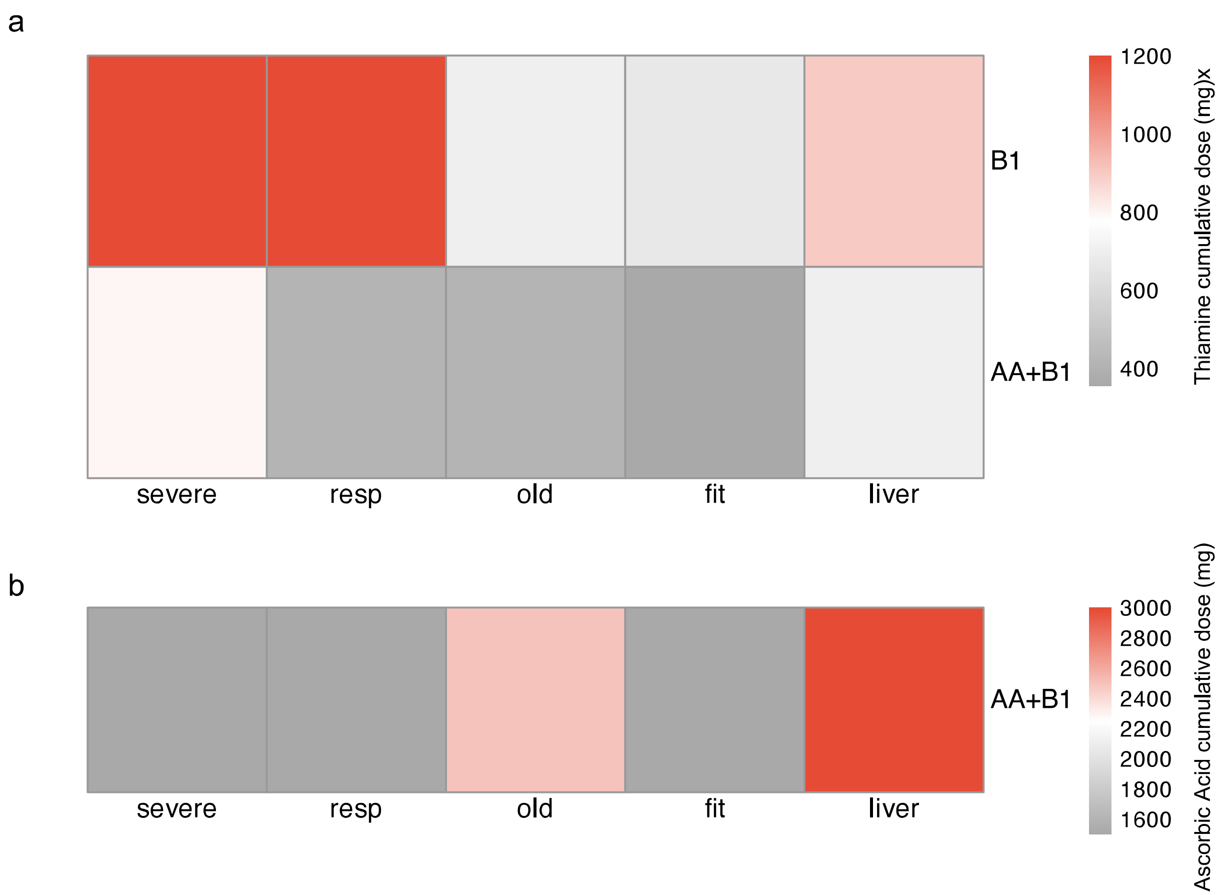

Supplementary Figure 4

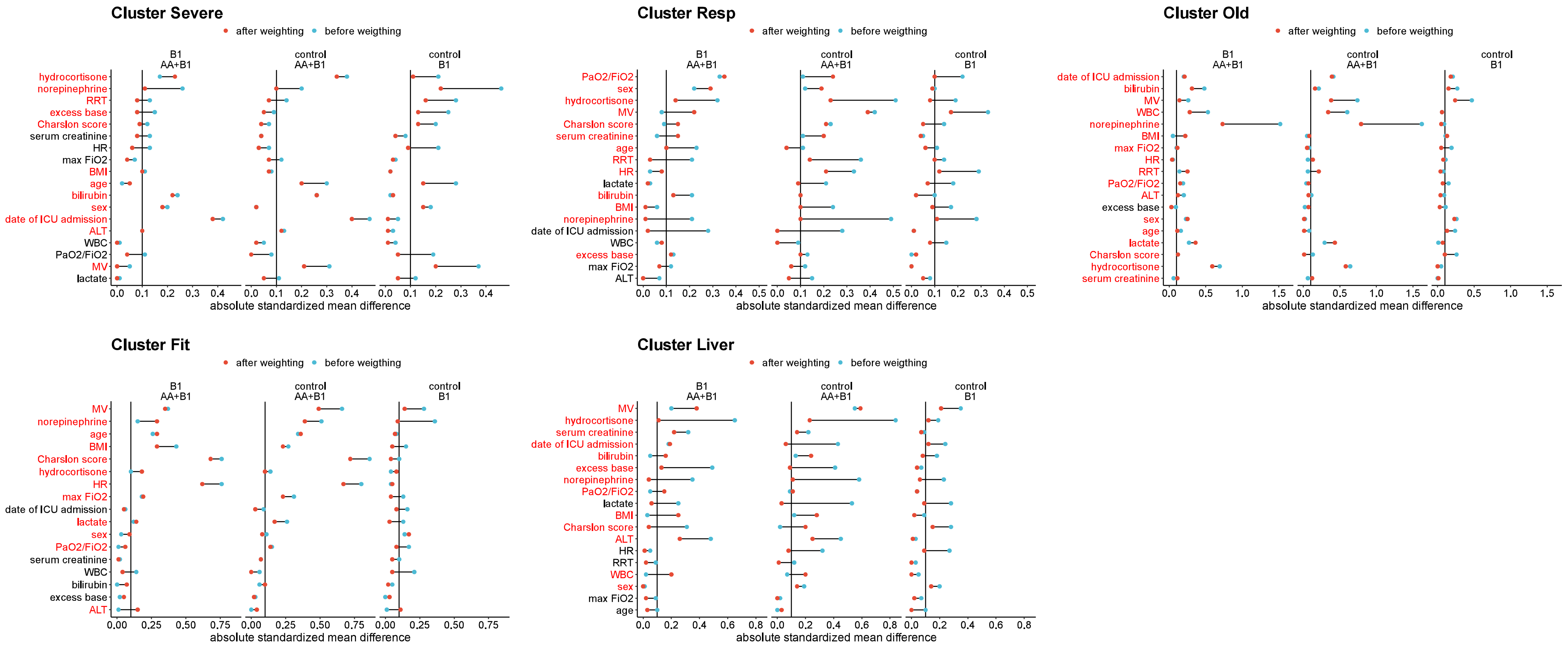

Supplementary Figure 5

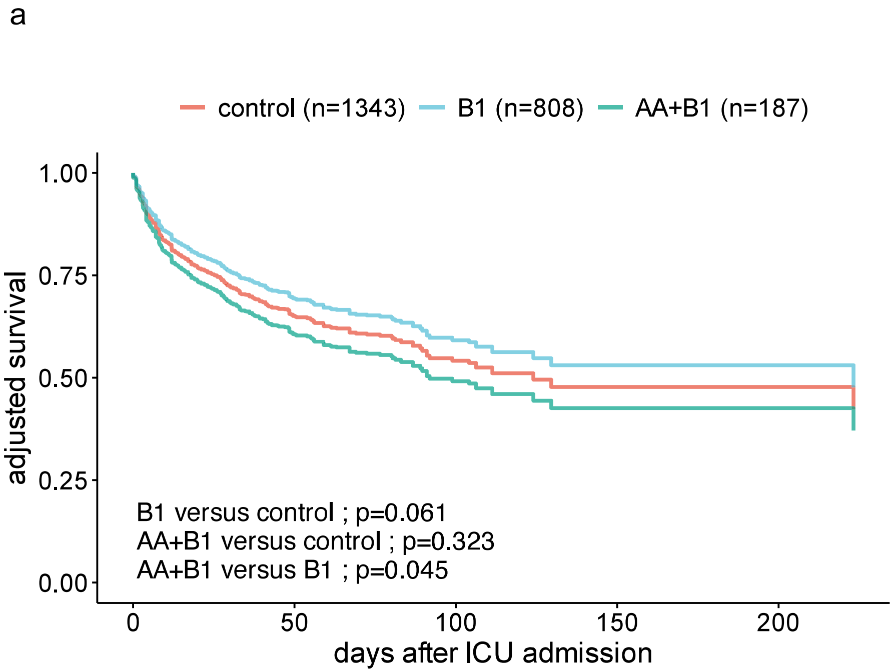

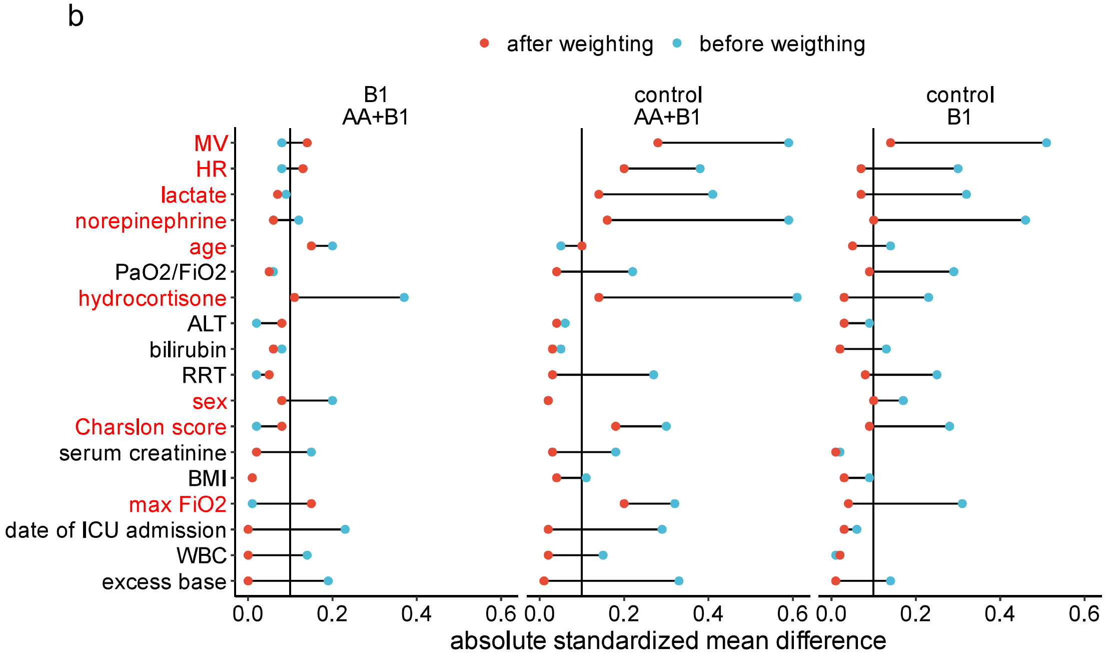

Supplement: Supplementary file 1 — Additional file1 (PDF 357 kb) [file 13054_2024_5188_MOESM1_ESM.pdf]
