# Supplementary material for: Differential effects of thiamine and ascorbic acid in clusters of septic patients identified by latent variable analysis
Source: Crit Care. 2024 Nov 29;28:396. doi: 10.1186/s13054-024-05188-4 (PMC11606082; doi:10.1186/s13054-024-05188-4)
Supplement: Supplementary file 2 — Additional file2 (DOCX 25 kb) [file 13054_2024_5188_MOESM2_ESM.docx]

**Supplementary *Figure 1 missing values****:* Dotplots showing the rate of missing values for every variable used.

*BMI Body Mass Index; COPD Chronic Obstructive Pulmonary Disease; DM Diabetes Mellitus; CKD Chronic Kidney Disease; RRT Renal Replacement Therapy; HR Heart Rate; MV Mechanical Ventilation; WBC White Blood Cell count, AST, Aspartate Aminotransferase; ALT Alanine Aminotransferase*

**Supplementary *Figure 2 variables balance across treatment groups****:* Dotplots showing the absolute standardized mean difference of each variable included in the propensity score, before (blue color) and after (red color) weighting across the treatment groups.

*Variables that still exhibit imbalance after weighting are highlighted in red and were further included as confounding variables in the Cox model.*

*BMI, Body Mass Index; RRT, Renal Replacement Therapy; HR, Heart Rate; MV, Mechanical Ventilation; WBC, White Blood Cell count; ALT, Alanine Aminotransferase*

**Supplementary Figure 3 Treatment regimens across clusters and groups:** heat map showing a) the median total cumulative dose of thiamine received across clusters in the B1 and AA+B1 groups and b) the median total cumulative dose of ascorbic acid received across clusters in the AA+B1 group.

**Supplementary *Figure 4 variables balance across treatment groups and for each cluster of patients****:* Dotplots showing the absolute standardized mean difference of each variable included in the propensity score, before (blue color) and after (red color) weighting and for each patient cluster. Variables that still exhibit imbalance after weighting are highlighted in red and were further included as confounding variables in the Cox model.

*BMI, Body Mass Index; RRT, Renal Replacement Therapy; HR, Heart Rate; MV, Mechanical Ventilation; WBC, White Blood Cell count; ALT, Alanine Aminotransferase*

***Supplemental Figure 5 sensitivity analyses without missing values imputation****:* ***a****) Survival curves showing the cumulative hospital survival vs time across the standard treatment, B1 and AA+B1 groups, after propensity score weighting and adjustment for residual unbalanced variables* ***b****)* Dotplots showing the absolute standardized mean difference of each variable included in the propensity score, before (blue color) and after (red color) weighting and for each patient subgroup. Variables that still exhibit imbalance after weighting are highlighted in red and were further included as confounding variables in the Cox model.

*BMI, Body Mass Index; RRT, Renal Replacement Therapy; HR, Heart Rate; MV, Mechanical Ventilation; WBC, White Blood Cell count; ALT, Alanine Aminotransferase*

**Supplementary Table 1.** Baseline characteristics of patients among treatments regimens.

BMI, Body Mass Index; SAPSII, Simplified Acute Physiological Score; MV, Mechanical Ventilation; WBC, White Blood Cells; RRT, Renal Replacement Therapy; ICU LOS, Intensive Care Unit Length of Stay; AKI, Acute Kidney Injury; AST, Aspartate Aminotransferase; ALT Alanine Aminotransferase; VFD, Ventilator Free Days, CKD Chronic Kidney Disease

|  | Control group (N=2183) | B1 group  (N=1054) | AA+B1 group  (N=228) | Total  (N=3465) | p value |
| --- | --- | --- | --- | --- | --- |
| **Patients’ characteristics** | | | | | |
| age | 69.0 (57.0, 77.5) | 66.0 (56.0, 74.0) | 69.0 (59.0, 76.0) | 68.0 (57.0, 76.0) | < 0.001 |
| Sex: Male | 1345 (61.6%) | 743 (70.5%) | 143 (62.7%) | 2231 (64.4%) | < 0.001 |
| BMI (kg/m2) | 24.8 (22.5, 28.3) | 24.7 (21.9, 28.4) | 25.0 (22.5, 28.7) | 24.8 (22.3, 28.4) | 0.417 |
| saps2 | 49.0 (38.0, 63.0) | 55.0 (44.0, 69.0) | 62.0 (51.0, 77.5) | 52.0 (40.0, 66.0) | < 0.001 |
| Charlson score | 3.0 (1.0, 6.0) | 4.0 (2.0, 6.0) | 4.0 (2.0, 7.0) | 3.0 (2.0, 6.0) | < 0.001 |
| Year of ICU admission | 2015.0 (2013.0, 2017.0) | 2016.0 (2013.0, 2019.0) | 2018.0 (2014.0, 2019.0) | 2015.0 (2013.0, 2018.0) | < 0.001 |
| **Underlying diseases** | | | | | |
| CKD | 619 (28.4%) | 177 (16.8%) | 35 (15.4%) | 831 (24.0%) | < 0.001 |
| diabetes | 405 (18.6%) | 216 (20.5%) | 40 (17.5%) | 661 (19.1%) | 0.362 |
| hypertension | 763 (35.0%) | 439 (41.7%) | 109 (47.8%) | 1311 (37.8%) | < 0.001 |
| heart_failure | 404 (18.5%) | 222 (21.1%) | 38 (16.7%) | 664 (19.2%) | 0.143 |
| chronic liver disease | 125 (5.7%) | 182 (17.3%) | 32 (14.0%) | 339 (9.8%) | < 0.001 |
| copd | 189 (8.7%) | 158 (15.0%) | 28 (12.3%) | 375 (10.8%) | < 0.001 |
| **At ICU admission** | | | | | |
| pH | 7.4 (7.3, 7.4) | 7.3 (7.2, 7.4) | 7.3 (7.2, 7.4) | 7.4 (7.3, 7.4) | < 0.001 |
| Base excess (mmol/L) | -5.2 (-8.5, -1.9) | -6.3 (-10.0, -2.4) | -7.0 (-11.3, -3.7) | -5.6 (-9.2, -2.1) | < 0.001 |
| HCO3 (mmol/L) | 19.5 (16.6, 22.6) | 19.0 (16.0, 22.5) | 18.6 (15.1, 21.6) | 19.2 (16.3, 22.5) | < 0.001 |
| Lactate (mmol/L) | 1.6 (1.0, 2.8) | 2.2 (1.3, 4.0) | 2.4 (1.6, 4.4) | 1.8 (1.1, 3.3) | < 0.001 |
| Bilirubine (mol/L) | 14.0 (9.0, 24.0) | 15.0 (10.0, 32.0) | 15.0 (10.0, 28.3) | 14.0 (9.0, 26.9) | < 0.001 |
| AST (UI/L) | 45.0 (26.0, 86.0) | 55.0 (31.0, 131.0) | 61.5 (31.0, 165.8) | 48.0 (27.0, 101.0) | < 0.001 |
| ALT (UI/L) | 31.0 (17.0, 59.5) | 33.0 (19.0, 68.3) | 36.5 (20.0, 85.5) | 32.0 (18.0, 62.7) | < 0.001 |
| Fibrinogen (g/L) | 4.8 (3.7, 6.0) | 4.4 (3.0, 5.7) | 4.6 (3.1, 5.9) | 4.7 (3.4, 5.9) | < 0.001 |
| Thrombocytes (G/L) | 173.0 (105.0, 252.0) | 155.0 (90.0, 243.0) | 160.0 (80.5, 259.2) | 169.0 (99.0, 250.0) | 0.021 |
| Serum creatinine (µmol/L) | 116.9 (77.0, 191.5) | 125.0 (79.0, 200.0) | 145.0 (95.0, 215.8) | 121.0 (78.0, 196.0) | < 0.001 |
| WBC counts (G/L) | 12.2 (7.2, 18.4) | 12.6 (6.5, 18.9) | 13.4 (6.7, 22.3) | 12.4 (6.9, 18.7) | 0.501 |
| **Within 24 hours following ICU admission** | | | | | |
| Maximal FiO2 (%) | 49.9 (31.2, 80.0) | 60.2 (40.0, 100.0) | 64.7 (44.7, 100.0) | 50.6 (35.0, 93.3) | < 0.001 |
| Minimal PaO2/FiO2 ratio | 200.3 (133.5, 269.3) | 159.7 (102.0, 234.6) | 164.4 (115.7, 244.1) | 187.0 (122.0, 259.5) | < 0.001 |
| Maximal PEEP (cmH20) | 7.0 (5.0, 8.3) | 8.0 (6.0, 10.0) | 8.0 (6.2, 10.0) | 7.0 (5.1, 9.0) | < 0.001 |
| Cumulative norepinephrine (mg) | 4.0 (0.1, 12.1) | 10.5 (2.1, 30.0) | 18.5 (6.8, 34.9) | 5.8 (0.7, 18.1) | < 0.001 |
| RRT | 151 (6.9%) | 159 (15.1%) | 39 (17.1%) | 349 (10.1%) | < 0.001 |
| MV | 745 (34.1%) | 634 (60.2%) | 158 (69.3%) | 1537 (44.4%) | < 0.001 |
| Hydrocortisone | 824 (37.7%) | 547 (51.9%) | 165 (72.4%) | 1536 (44.3%) | < 0.001 |
| **Outcomes** | | | | | |
| ICU LOS (days) | 2.6 (1.3, 5.5) | 5.6 (2.3, 11.4) | 4.0 (2.7, 7.4) | 3.3 (1.6, 7.5) | < 0.001 |
| Hospital LOS (days) | 16.0 (7.0, 31.0) | 20.0 (9.2, 35.2) | 15.0 (5.0, 30.7) | 17.0 (7.7, 33.0) | < 0.001 |
|  |  |  |  |  | < 0.001 |
| No AKI | 1395 (63.9%) | 404 (38.3%) | 92 (40.4%) | 1891 (54.6%) |  |
| KDIGO1 | 222 (10.2%) | 107 (10.2%) | 20 (8.8%) | 349 (10.1%) |  |
| KDIGO2 | 302 (13.8%) | 220 (20.9%) | 58 (25.4%) | 580 (16.7%) |  |
| KDIGO 3 | 264 (12.1%) | 323 (30.6%) | 58 (25.4%) | 645 (18.6%) |  |
| VFD at day28 (days) | 23.1 (0.0, 26.8) | 17.2 (0.0, 24.0) | 14.4 (0.0, 25.1) | 20.8 (0.0, 26.0) | < 0.001 |
| ICU mortality (%) | 316 (14.5%) | 206 (19.5%) | 66 (28.9%) | 588 (17.0%) | < 0.001 |
| Hospital mortality (%) | 474 (21.7%) | 294 (27.9%) | 91 (39.9%) | 859 (24.8%) | < 0.001 |
| 28 days mortality (%) | 436 (21.4%)  144 missing | 276 (27.1%)  34 missing | 85 (37.8%)  3 missing | 797 (24.3%)  181 missing | < 0.001 |

Data were shown as number (%) or median (IQR)

**Supplementary Table 2**

*Treatment regimens in groups of treatments.*

|  | Control group  (n=2183) | B1 group  (n=1054) | AA+B1 group  (n=228) |
| --- | --- | --- | --- |
| **B1** |  |  |  |
| Total cumulative dose (mg), median (IQR) | - | 900 (400;2500)) | 600 (300; 1533) |
| Total treatment duration, (days) median (IQR) | - | 3.4 (1.0;7.0) | 2.0 (1.0; 3.0) |
| **AA** |  |  |  |
| Total cumulative dose (mg), median (IQR) | - | - | 1500 (1250;4875) |
| Total treatment duration, (days) median (IQR) | - | - | 1.2 (1.0; 2.0) |
| **Hydrocortisone use n (%)** | 824 (37.7) | 547 (51.9) | 165 (72.4) |

Data were shown as number (%) or median (IQR)

**Supplementary Table 3**

*Treatment regimens across patient clusters.*

|  | Cluster Severe  (N=554) | Cluster Resp (N=816) | Cluster Old  (N=653) | Cluster Fit  (N=810) | Cluster Liver  (N=632) | Total (N=3465) | p value |
| --- | --- | --- | --- | --- | --- | --- | --- |
| **Control group** | (N=242) | (N=437) | (N=491) | (N=619) | (N=394) | (N=2183) |  |
| Hydrocortisone use n(%) | 179 (74.0%) | 194 (44.4%) | 182 (37.1%) | 119 (19.2%) | 150 (38.1%) | 824 (37.7%) | < 0.001 |
| **B1 group** | (N=245) | (N=312) | (N=127) | (N=167) | (N=203) | (N=1054) |  |
| Total B1 cumulative dose (mg) | 1200 (550, 3000) | 1200 (500, 3300) | 700 (300, 1563) | 666 (300, 1550) | 900.0 (472 210) | 900 (400, 2500) | < 0.001 |
| Toal B1 duration (days) | 5.0 (1.0, 9.0) | 5.0 (3.0, 9.0) | 2.0 (1.0, 5.0) | 1.9 (1.0, 4.0) | 2.8 (1.0, 5.0) | 3.4 (1.0, 7.0) | < 0.001 |
| Hydrocortisone use n(%) | 202 (82.4%) | 169 (54.2%) | 44 (34.6%) | 35 (21.0%) | 97 (47.8%) | 547 (51.9%) | < 0.001 |
| **AA+B1 group** | (N=67) | (N=67) | (N=35) | (N=24) | (N=35) | (N=228) |  |
| Total B1 cumulative dose (mg) | 800 (300, 1905) | 414 (300, 1500) | 414 (300, 1200) | 355 (205, 903) | 700 (30, 1600) | 600 (300, 1533) | 0.336 |
| Total B1 duration (days) | 2.0 (1.0, 4.0) | 2.0 (1.0, 3.0) | 2.0 (1.0, 3.0) | 2.0 (1.0, 3.2) | 1.5 (1.0, 3.2) | 2.0 (1.0, 3.0) | 0.356 |
| Total AA cumulative dose (mg) | 1500 (1250, 6250) | 1500 (1250, 4500) | 2500 (1250, 5250) | 1500 (1000, 1875) | 3000 (1375, 6750) | 1500 (1250, 4875) | 0.128 |
| Total AA duration (days | 1.8 (1.0, 2.1) | 1.7 (1.0, 2.0) | 1.5 (1.0, 2.0) | 1.0 (1.0, 2.0) | 1.0 (1.0, 1.9) | 1.2 (1.0, 2.0) | 0.279 |
| Hydrocortisone use n(%) | 60 (89.6%) | 47 (70.1%) | 24 (68.6%) | 6 (25.0%) | 28 (80.0%) | 165 (72.4%) | < 0.001 |

Data were shown as number (%) or median (IQR
